# A 16-Year-Old Male with Thoracic Compression following Posterior Spinal Instrumentation and Fusion for Marfan-Associated Syndromic Scoliosis

**DOI:** 10.1155/2020/6617028

**Published:** 2020-12-10

**Authors:** Mason Uvodich, Ross Barman, Adam Reitz, Matthew Sexton

**Affiliations:** ^1^Mayo Clinic Department of Orthopaedic Surgery, USA; ^2^Mayo Clinic Department of Anesthesiology and Perioperative Medicine, USA; ^3^Kentucky College of Osteopathic Medicine, USA; ^4^Department of Critical Care Medicine, USA

## Abstract

**Introduction:**

Marfan syndrome is an autosomal dominant disorder caused by a mutation in the *FBN1* gene which affects connective tissue. The features of Marfan syndrome include many musculoskeletal abnormalities which require orthopaedic surgical intervention. Given the expansive phenotypic variations and comorbidities associated with Marfan syndrome, knowledge of perioperative risk factors and potential complications is essential.

**Case:**

In this case report, the authors describe a patient with Marfan syndrome who underwent spinal instrumentation and fusion from T3 to L4 for correction of syndromic scoliosis. The patient had a complicated perioperative course requiring significant fluid resuscitation and vasoactive medications to support blood pressure. He required intensive care unit level care for continued hemodynamic instability despite resuscitation in the postoperative period. Common causes of postoperative hypotension such as hypovolemic shock, sepsis, ongoing hemorrhage, and prolonged effects of anesthesia were diagnostically ruled out. Ultimately, the patient's refractory hypotension was determined to be from mechanical compression, both from prolonged intraoperative prone positioning exacerbated by pectus excavatum and from the surgically corrected spine decreasing the diameter of his thoracic cavity (as referenced by his postoperative Haller index).

**Conclusion:**

Mechanical compression of thoracic contents as a result of a worsening chest wall deformity can be a complication of spinal deformity correction.

## 1. Introduction

Marfan syndrome affects an estimated 1 in 10,000 people and is inherited in an autosomal dominant fashion. It is the result of a mutation in the extracellular matrix protein fibrillin-1 gene found on chromosome 15 which alters connective tissues [1]. Orthopaedic surgery is common in patients with Marfan syndrome because 60% will have scoliosis, and of those with scoliosis, an estimated 25% to 50% of patients will require surgical intervention for correction after failing conservative measures [2]. Marfan syndrome has a spectrum of manifestations that should be considered in the perioperative and ICU settings, such as predispositions to mitral valve prolapse, spontaneous pneumothorax, extreme scoliosis compressing other organs, sternal deformities, dilation of the ascending aorta, and aortic aneurysm. In the following case report, we present a unique case involving a pediatric patient with Marfan syndrome who presented to the ICU with refractory undifferentiated shock following posterior spinal instrumented fusion (PSIF) and deformity correction.

## 2. Case Description

A 16-year-old male with a past medical history of Marfan syndrome with severe pectus excavatum, significant scoliosis, and generalized anxiety disorder presented for PSIF from T3 to L4 to correct his scoliosis deformity. Preoperative evaluation demonstrated a left lumbar curve of 64 degrees and lumbar kyphosis of 14 degrees. His curve corrected to 40 degrees with a fulcrum bend. Evaluation 1 year prior to the surgery revealed no cardiovascular abnormalities with a normal ejection fraction of 56%.

Induction of anesthesia and intubation were uncomplicated. His preprocedure vitals were stable and within a normal range. The patient was placed in a prone position. Due to the ongoing hemodynamic instability and need for abundant amounts of vasopressor support, the chest pads were repositioned which improved his hypotension for a time. The surgical aspects of the procedure proceeded uneventfully. The patient had transverse process pedicle hooks placed bilaterally at T3 and had navigated pedicle screws placed T4-L4. Facetectomies were performed at each level and a Cortrel-Dubousset derotation maneuver was performed to correct the scoliosis. Sequential distraction and compression was performed to correct the deformity in combination with in situ rod bending in both the coronal and the sagittal plane. Throughout the procedure, he had a significant vasopressor requirement. The patient received adequate crystalloid and colloid in addition to 4 units of fresh frozen plasma and approximately 600 cc of cell saver. The case lasted 10 hours and was complicated with 1.4 L of blood loss.

At the conclusion of the case, the patient was flipped supine and transferred to the postanesthesia care unit (PACU) intubated and on mechanical ventilator support in hopes to later extubate once his anesthetic dissipated. He was slow to emerge from anesthesia, and initial vital signs showed significant hypotension (systolic blood pressure 66 mmHg with a mean arterial pressure (MAP) of 54) and tachycardia into the 140s. The patient was started on phenylephrine and was transferred to the Surgical Intensive Care Unit (SICU) for ongoing management.

**Figure 1:**
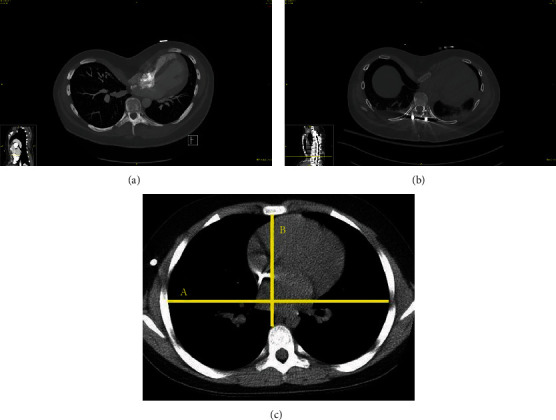
Comparison of preoperative and postoperative CT chest. Preoperative CT chest had a Haller index of 8.7. The anterior-posterior diameter minimum preoperatively was 3 cm (a). Postoperative CT chest had a Haller index of 11.3 (b). The anterior-posterior diameter minimum postoperatively was 2.3 cm. Example of a normal chest CT Haller index (c), with lines demonstrating measurements of the Haller index (ratio of A/B) [6].

Over the next few hours, the patient became interactive and was extubated uneventfully; however, his hypotension continued despite phenylephrine which resulted in a transition to a norepinephrine infusion. His vitals continued to demonstrate a narrowed pulse pressure with hypotension and tachycardia, concerning for cardiac tamponade and/or active postsurgical bleeding. Laboratory investigations demonstrated a hemoglobin of 12.5 g/dL, international normalized ratio (INR) of 1.6, activated partial thromboplastin (APP) time of 30, and a fibrinogen of 155 mg/dL which improved to 187 mg/dL without intervention. He was given fluid boluses and continued on norepinephrine with minimal improvement. His initial postoperative labs were significant for a lactate of 3.6 mmol/L and a creatinine (Cr) of 0.8 mg/dL. His lactate peaked at 5.0 mmol/L. Given continued concern for myocardial depression, tamponade physiology, or active bleeding, he was assessed with an emergent formal bedside transthoracic echocardiogram and CT angiogram of the chest and abdomen. These demonstrated no active sources of bleeding, no pneumothoraces, and a relatively unfilled, hyperdynamic left ventricle with a postoperative Haller index that increased from 8.7 to 11.3 (Figure 1).

**Figure 2:**
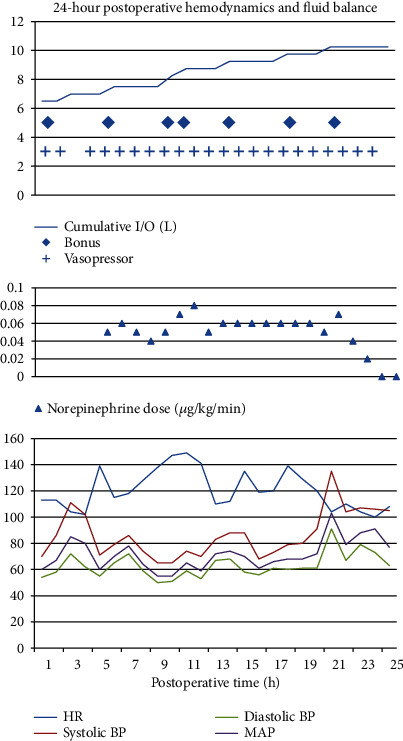
Postoperative hemodynamics and fluid balance data include measurements of blood pressure (BP) in millimeters of mercury (mmHg) and include systolic BP, diastolic BP, and mean arterial pressure (MAP). Heart rate (HR) is presented in beats per minute. Cumulative input and output (I/O) is for the patient's entire hospital course and is presented in liters (L). Starting at one hour postoperative, the patient was approximately 6 liters positive. Crystalloid and colloid boluses are designated under the same title “bolus,” which is identified by diamonds in the graphs. Vasopressor refers to any vasopressive medication given in the postoperative period and is designated by crosses. Vasopressin and ephedrine were given in the early postoperative period followed by a transition to norepinephrine alone at approximately 4 hours postoperative. Norepinephrine was administered in micrograms per kilogram per minute (*μ*g/kg/min) and was stopped approximately 23 hours postoperative.

**Figure 3:**
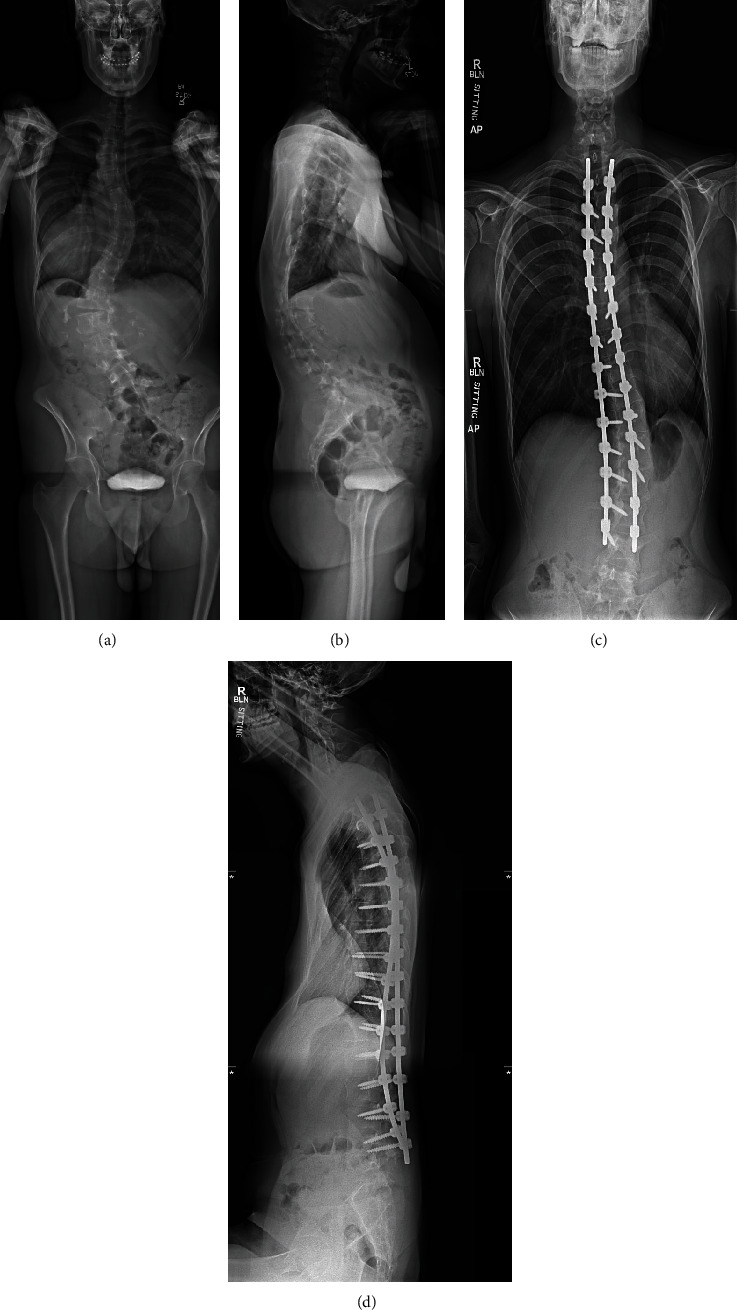
Preoperative and postoperative radiographs. The top two images are the preoperative standing posterior-anterior (a) and lateral (b) plain radiographs of the entire spine. The bottom two images are six-month postoperative standing anterior-posterior (c) and lateral (d) plain radiographs of the spine.

Over the next several hours, surveillance labs showed an evolving acute kidney injury and signs of global hypoperfusion as his lactate continued to rise. We suspected that his pectus excavatum had compromised his cardiac function by mechanical obstruction and that this compromise was exacerbated by significant intraoperative blood loss, fluid deficit, and residual effects of his anesthetic. Cardiothoracic surgery was consulted and gave consideration to immediate surgical intervention should the patient not improve with medical management. We utilized fluid boluses and vasopressor support over the course next 24 hours (Figure 2). He slowly improved hemodynamically. His acute kidney injury, lactate, and urine output improved, and he was ultimately transferred out of the ICU on postoperative day two without additional surgical intervention. Preoperative and postoperative radiographs demonstrating the spinal instrumentation and deformity correction are provided (Figure 3).

## 3. Discussion

Adolescent scoliosis is classified as either idiopathic, neuromuscular, congenital, or syndromic. Marfan syndrome is a cause of syndromic scoliosis. The estimated prevalence of scoliosis in patients with Marfan syndrome varies but is greater than 30%, if not much higher [3]. Complication rates among patients with Marfan syndrome undergoing corrective procedures are typically higher than those among patients without Marfan syndrome [1, 4]. A retrospective review of a national registry found the most common complications to be pulmonary- or implant-related [4]. Kurucan et al. [4] also found that patients with Marfan syndrome did not have higher odds of cardiac complications compared to matched controls, although pediatric Marfan patients had a greater blood loss compared to controls. Perhaps the age or noncardiac surgery limits the cardiac complications in these patients, especially considering Marfan patients' known predisposition to aortic root and valvular pathology. In addition, there is limited literature describing intraoperative or postoperative sequelae of chest wall deformity particularly in scoliosis.

Intraoperative positioning is especially important for extended duration surgical procedures and likely played a role in our patient's refractory postoperative hypotension. While prone positioning has well-known complications of endotracheal tube displacement and ocular injury secondary to improper padding placement, abdominal compartment syndrome and reduced cardiac index are also frequently encountered in the postoperative course. Prone positioning during surgery affects the cardiac index by reducing stroke volume, and this risk is exacerbated by blood loss, intraoperative fluid shifts, and anatomic deformities that inherently obstruct cardiac output such as pectus excavatum or thoracic lordosis [5]. Mechanical obstruction is not relieved by vasopressors or fluid resuscitation; this is likely why our patient suffered from significant intraoperative hypotension.

Pectus excavatum is a deformity of the chest wall characterized by a posterior depression of the sternum and the adjacent costal cartilage. The deformity varies in depth by vertebral level [6]. The severity of pectus excavatum is graded via the Haller index which is the quotient of the transverse diameter of the chest wall divided by the smallest possible distance from the posterior aspect of the sternum to the anterior vertebral body [7]. A value > 3.5 is considered severe [8]. Our patient's Haller index was approximately 8.7 preoperatively which increased postoperatively to 11.3 (Figure 1). Without evidence of other causes for our patient's hemodynamic instability, we considered this as the leading etiology in our patient.

Pectus excavatum is common in patients with Marfan syndrome (as well as carinatum) and occurs at a rate of approximately 30% [9]. There is a paucity of literature describing the effects of pectus excavatum on the perioperative complications of posterior spinal fusion in patients with Marfan syndrome. Overall, the rates of major complications in adolescent spine surgery for various etiologies are approximate at 3-20% [10, 11]. Main risk factors for major complications appear to be nonweight-bearing status, intraoperative blood loss, and preexisting pulmonary compromise [11]. Sternal compression of the heart with prone positioning has been reported to cause intraoperative hypotension [12]. A case report on a patient similar to ours who underwent elective surgical correction of his pectus excavatum following intolerance of prone positioning had a good outcome [13]. A recent study of 20 patients with pectus excavatum and scoliosis undergoing a corrective spine procedure within the thoracic spine had their pectus increase in severity in 11/20 patients [14]. Pectus excavatum has been shown to affect cardiac rotation and on occasion compress large vessels with prone positioning which is demonstrated in our patient's CT scan (Figure 1) [15, 16].

What is curious about our case is the prolonged hypotension after surgery. This initially suggested the more common causes of postoperative hypotension such as ongoing hemorrhage, fluid deficit secondary to blood loss or insensible losses, or myocardial stunning (from intraoperative positioning) [13, 17, 18]. However, upon further evaluation and based off of the patient's clinical course, we feel the patient's comorbid chest wall deformity, worsening Haller index, and prolonged prone positioning suggest an initial and continued mechanical compression of his thoracic contents that was made worse after his corrective surgery.

The effects of pectus excavatum on contributions to perioperative complications in Marfan patients are largely unstudied. Fortunately, our patient's hemodynamic instability improved with volume resuscitation and temporary vasoactive support, but one should also always consider additional causes within this patient population such as acute postoperative mechanical cardiac compression which warrants surgical evaluation.

## Data Availability

There are no available data supporting the results of this study as it is a case report.
